# Perceptual grouping, not covert attention, drives the connectedness effect in the ANS.

**DOI:** 10.1007/s00426-026-02255-z

**Published:** 2026-02-24

**Authors:** Andrea Adriano, Michaël Vande Velde

**Affiliations:** 1 Department of Psychology, Sapienza University of Rome, Via Dei Marsi 78, Rome, 00185 Italy; 2 Laboratoire Cognition Langage et Développement, Université Libre de Bruxelles, Bruxelles, Belgium

**Keywords:** Numerosity perception, Connectedness effect, Perceptual grouping, Covert attention, Approximate Number System (ANS)

## Abstract

A robust finding in numerical cognition is that connecting items within an array leads to systematic underestimation of numerosity. This provides evidence that approximate numerosity perception relies on discrete objects rather than on continuous variables (e.g., total area, density, convex hull). While this *connectedness effect* is often attributed to perceptual grouping, an alternative interpretation is that connected items may capture covert attention, thereby biasing the sampling of visual information. We tested these competing accounts by combining a numerosity estimation task with a target-detection task modeled after Posner’s cueing paradigm. On each trial, participants viewed dot arrays (14–20 items) that included two red lines, either connecting a pair of dots or terminating near unconnected dots. A target diamond could appear either near (congruent) or far (incongruent) from the quadrant containing the red lines. Participants first performed a go/no-go detection task, then estimated the array numerosity. Replicating prior work, connected arrays were consistently underestimated relative to unconnected ones. Crucially, detection performance showed no evidence of attentional capture: reaction times and accuracy did not differ as a function of connectedness or target position. These findings demonstrate that the underestimation effect cannot be attributed to *covert* attentional allocation. Instead, they support the view that perceptual grouping—rather than attentional biases—drives the connectedness effect in the Approximate Number System. More broadly, our results strengthen the case for segmentation-based mechanisms as a critical foundation of visual number perception.

## Introduction

There is broad consensus that humans and many animal species share a fundamental neurocognitive mechanism — the *Approximate Number System* (ANS) — which enables the extraction of non-symbolic, approximate numerical representations from large sets of objects (Dehaene et al., 1998; Nieder, 2016). Evidence for this system has been documented across a variety of species (e.g., Agrillo et al., 2009; Brannon & Terrace, 1998; Ditz & Nieder, 2015) and in early stages of human development (e.g., Brannon et al., 2004; Xu & Spelke, 2000; Xu et al., 2005). From an evolutionary perspective, this underscores the adaptive value of a specialized numerical mechanism for survival-relevant tasks, such as comparing group sizes of conspecifics or rivals, or selecting the larger food source (e.g., Agrillo et al., 2012; Benson-Amram et al., 2011; Perdue et al., 2012; Piantadosi & Cantlon, 2017).

A hallmark behavioral property of the ANS is its compliance with Weber’s law, observed consistently across cultures, developmental stages, and species (e.g., Whalen et al., 1999). Weber’s law states that the just-noticeable difference between two stimuli scales with their magnitude, or more strictly, that discrimination accuracy depends on the ratio of their intensities (Brus et al., 2019; Dehaene, 2003). This has been extensively investigated using rapid numerical comparison tasks, in which participants choose the larger or smaller of two briefly presented arrays. In such tasks, error rates and reaction times increase as the numerical ratio approaches unity, indicating that non-symbolic number processing follows Weber’s law (e.g., Revkin et al., 2008).

At the neural level, numerosity-selective responses have been identified in the parietal cortex of both humans and macaques (e.g., Castelli et al., 2006; Harvey et al., 2013; Nieder & Miller, 2004; Piazza et al., 2004). More recent findings suggest that a wider network, including early visual areas, may also contribute to numerosity processing (DeWind et al., 2019; Fornaciai et al., 2017; Fornaciai & Park, 2018; Park et al., 2015; Van Rinsveld et al., 2020).

Although the existence of the ANS is widely accepted, the precise visual computations it relies on remain debated. Some models posit that numerosity is obtained through a segmentation-and-individuation process that counts discrete items regardless of their physical properties such as shape, size, or position (Burr & Ross, 2008; Dehaene & Changeux, 1993; Stoianov & Zorzi, 2012; Verguts & Fias, 2004). Strong support for this view comes from *numerosity adaptation* studies, where prolonged exposure to a set of a given numerosity biases the perceived numerosity of subsequent sets in the adapted location (Burr & Ross, 2008; Thompson & Burr, 2009). Typically, adaptation to a large number reduces the perceived numerosity of a smaller set, and adaptation to a small number increases the perceived numerosity of a larger set (Aagten-Murphy & Burr, 2016; Aulet & Lourenco, 2023). Such adaptation effects, observed across sensory modalities, have led to the proposal that numerosity functions as a primary sensory attribute, akin to colour, motion, or spatial frequency (Arrighi et al., 2014; Burr et al., 2025, but see Yousif et al., 2024, 2025).

However, other psychophysical evidence challenges the notion of numerosity as a primary sense (e.g., Durgin, 2008). Alternative accounts suggest that numerical judgments are derived indirectly from continuous visual features correlated with numerosity, such as total occupied area, density, or luminance (Allik & Tuulmets, 1991; Dakin et al., 2011; Durgin, 2008; Gebuis & Reynvoet, 2012a, b, c). The *occupancy model* (Allik & Tuulmets, 1991) proposes that numerosity is estimated by summing the “virtual” area occupied by items, with greater overlap leading to underestimation. Numerous studies have shown that item size, total surface area, convex hull, and density can all bias numerosity discrimination (e.g., Gebuis & Reynvoet, 2012a; Hurewitz et al., 2006; Katzin et al., 2020). Given that it is physically impossible to create two sets differing in numerosity yet identical in all continuous features, it has been argued that the visual system may integrate one or more of these correlated cues to infer number, without requiring a dedicated discrete-number mechanism (Gebuis et al., 2016; Leibovich et al., 2017).

Consistent with this view, Durgin (2008) highlighted the role of texture-density statistics, while Dakin et al. (2011) proposed that density estimation could be achieved by analysing spatial-frequency content. Other biologically inspired models suggest that “contrast energy” could serve as a proxy for numerosity (Morgan et al., 2014). Still, recent work indicates that a dedicated density-processing system may operate for very large numerosities (≈ 100 items), where crowding prevents individual segmentation (Anobile et al., 2014, 2016; cf. Portley et al., 2019). In such cases, Weber fractions remain constant at low numerosities but decrease with the square root of numerosity beyond a critical threshold (Anobile et al., 2014). This pattern suggests that, at least for moderate numerosities, visual number processing depends on item segmentation and cannot be fully reduced to texture-density mechanisms (Anobile et al., 2015; Pomè et al., 2019).

Therefore, several investigations have employed innovative experimental approaches to directly test competing explanations, making use of visual illusions such as size illusions, illusory contours (ICs) and connectedness-based grouping (e.g., Adriano et al., 2021, 2022; Franconeri et al., 2009; Picon et al., 2019). Consistent with the Gestalt principle of uniform connectedness (Palmer & Rock, 1994), a number of studies have reported that perceived numerosity is systematically underestimated when elements within an array are linked either by actual connecting lines (Anobile et al., 2017; Fornaciai & Park, 2018; Franconeri et al., 2009; He et al., 2009, 2015; Pomè et al., 2022) or by task-irrelevant illusory connections (Adriano et al., 2021, 2022; Adriano & Ciccione, 2024; Kirjakovski & Matsumoto, 2016), even when low-level visual attributes are held constant across connectedness conditions. Collectively, these results indicate that visual segmentation processes are central to the extraction of discrete numerical information.

However, connected objects differ from unconnected ones not only in their grouping status but also in their visual salience: their “multi-part” structure can make them pop out from surrounding items, potentially attracting more attention to their location (He et al., 2009). Such attentional capture could reduce the processing time available for other items in the display (e.g., in the uncued locations), leading to their partial or total exclusion from the numerosity encoding. In this view, the observed numerical underestimation reported with the connectedness illusion might arise from differences in attentional allocation rather than from perceptual grouping per se.

Previous studies have attempted to control for *overt* attentional shifts by using very brief stimulus presentations (e.g., 50 ms) to prevent eye movements (He et al., 2009). Paradoxically, these studies found that the underestimation effect was even larger at short exposure durations. Therefore, this leaves open the possibility that *covert* attention — the selective processing of visual information without eye movements (Posner, 1980) — could still play a role. If connected items automatically attract covert attention, they might draw processing resources away from other regions of the display, thereby biasing numerosity judgments through changes in the effective sampling of the scene.

To the best of our knowledge, no previous study has accounted for this potential alternative explanation of the connectedness effect. To address this gap, we designed a novel task inspired by Posner’s classic attentional paradigm (1980).

Similarly to the classic Posner cueing task, our paradigm required participants to identify a briefly flashed target (e.g., a diamond) that appeared either near (congruent) or far (incongruent) from the spatial position of two red lines acting as exogenous cues. The red lines were intermingled with a variable number of dots and could either connect or remain unconnected to two of them. Participants performed a go/no-go detection task, responding as quickly as possible when the target appeared. Immediately afterward, they reported the total number of objects displayed on the screen.

We reasoned that, given the salience of the red line cues (connected or unconnected), they should pop out from the surrounding items and automatically attract covert attention to their location. Consequently, detection times were expected to be faster when the target appeared near (congruent) rather than far (incongruent) from the quadrant containing the red lines, resulting in a significant main effect of target position.

Furthermore, according to the covert attention hypothesis, a red line connecting multiple dots forms a distinct perceptual object (a “dumbbell object”) that automatically attracts attention. When this multipart object is located near the target, attention is strongly drawn to that location, facilitating faster detection of the diamond. Conversely, if the object is positioned farther from the target, it still captures attention, but less effectively with respect to the target, producing only a moderate effect on detection. By contrast, a non-connected line does not form a coherent object and therefore exerts a weaker influence on attention, regardless of its spatial position. Consequently, the difference in attentional capture between near and far locations is predicted to be substantially larger for connected lines than for non-connected lines, giving rise to the expected interaction between spatial position and connectedness. In this case, the underestimation effect could be attributed to attentional allocation rather than to perceptual grouping per se.

Conversely, if no main effects or interactions emerged in the detection task, but connected objects were still underestimated in the estimation task, this would suggest that both connected and unconnected objects engage covert attention similarly, and that the underestimation effect cannot be primarily explained by attentional biases.

## Methods

### Participants

An a priori power analysis was conducted using G*Power 3.1 (Faul et al., 2009) to estimate the required sample size. Based on previous findings from a study adopting a comparable design (Adriano & Vande Velde, 2025b), which reported a partial eta-squared (η²ₚ) of 0.41 for the Connectedness factor, we calculated that a minimum of 18 participants would be necessary to achieve 80% power in a repeated-measures ANOVA with four conditions (Close Target Connected/Unconnected and Far Target Connected/Unconnected), assuming α = 0.05. Nineteen participants were recruited for the present study (mean age = 19.52 years, SD = 1.42; 16 females; 17 right-handed participants). All participants had normal or corrected-to-normal vision. The study was approved by the local Ethics Committee.

### Stimuli and design

The stimuli were generated offline using a custom Python/PsychoPy script (Peirce, 2007) and presented on a 19” LCD monitor (1280 × 960 pixels; 60 Hz) connected to a standard desktop computer. Each stimulus consisted of a 12 × 12 black grid (cell size: 22 px; line width: 2 px; RGB: −1, − 1, −1). Within the grid, a variable number of white filled dots (radius: 6 px; RGB: 1, 1, 1) with a thin black outline (1 px; RGB: −1, − 1, −1) were placed on the grid intersections. The number of dots varied across four levels: 14, 16, 18, and 20.

Each stimulus also included two parallel red lines oriented at 45° (width: 2 px; RGB: 1, 0, 0), randomly positioned in one of four predefined quadrants (upper-left, upper-right, lower-left, lower-right). The position of the red lines was counterbalanced across quadrants. To manipulate connectedness, in half of the stimuli two pairs of dots were positioned at the endpoints of the lines, whereas in the other half the same spatial configuration was preserved, but the dots were placed on the opposite sides of the line terminations.

**Fig. 1:**
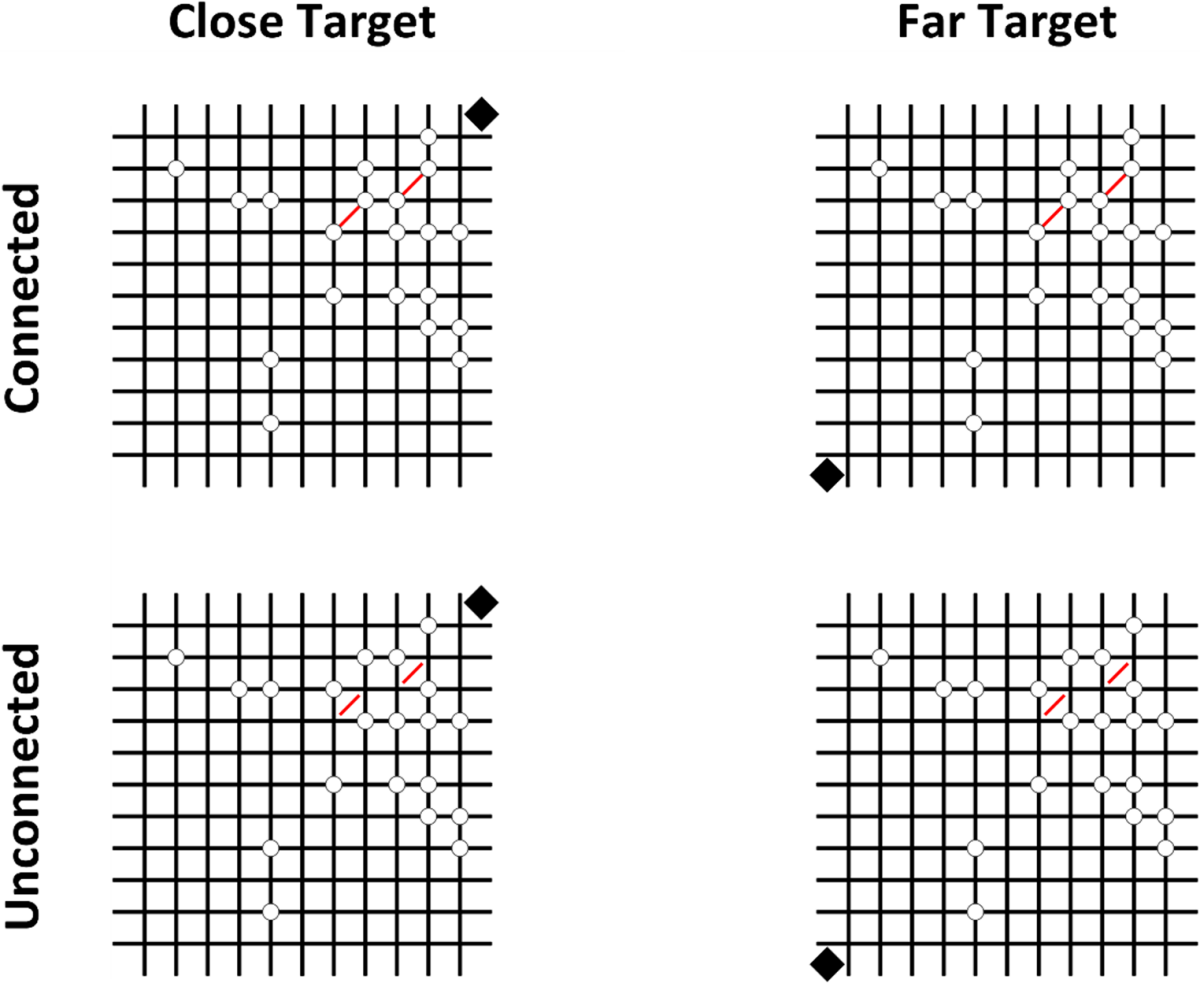
Stimuli consisted of dot patterns (14–20 dots) displayed on a 12 × 12 grid, with two red parallel lines randomly placed in one quadrant. A black diamond target appeared in 50% of the trials, either near or far from the line quadrant

In 50% of the trials, a black diamond (20 × 20 px; RGB: −1, − 1, −1) appeared in one of the four grid corners, while in the remaining trials the target was absent. That is, exogenous cueing followed a standard 50:50 cue validity (Klein et al., 2009). Target position was counterbalanced across quadrants and could be either near (same corner) or far (opposite corner) relative to the quadrant containing the two lines (Fig. 1).

Overall, the experiment comprised 64 conditions when the target was present (Go trials: 4 numerosities × 2 connectedness levels × 2 target positions × 4 quadrants) and 32 conditions when it was absent (No-Go trials: 4 numerosities × 2 connectedness levels × 4 quadrants). Each combination of quadrant position and numerosity was repeated 8 times (2 target positions × 2 connectedness levels × 2 Go/No-Go conditions). Each condition was presented with two unique visual patterns, resulting in a total of 256 stimuli.

### Procedure

The experiment was conducted in a quiet, dimly lit room, and participants were tested individually. The general procedure was explained to each participant before the experiment began, and detailed instructions were also presented on the screen. Participants were comfortably placed at about 50 cm from the screen.

Participants performed a dual task. First, they completed a *go/no-go detection task*: they had to press the space bar as quickly as possible if a target appeared in one of the corners (go) and withhold their response if it did not (no-go). Second, to ensure that their attention remained focused on the center of the display rather than the corners, participants also completed an *estimation task*, reporting as accurately as possible the number of objects in the array by typing their estimate on the numerical keypad of a standard PC keyboard. No information about the connectedness of the stimuli was provided to the participants. The participants were, in any case, instructed to provide a numerical estimate between 1 and 40. To familiarize participants with the procedure, the experimental phase was preceded by a short practice session (24 trials) with partial feedback (e.g., negative feedback was given only if the target was missed, and positive feedback was provided only if the exact number of items was reported in the estimation task).

**Fig. 2:**
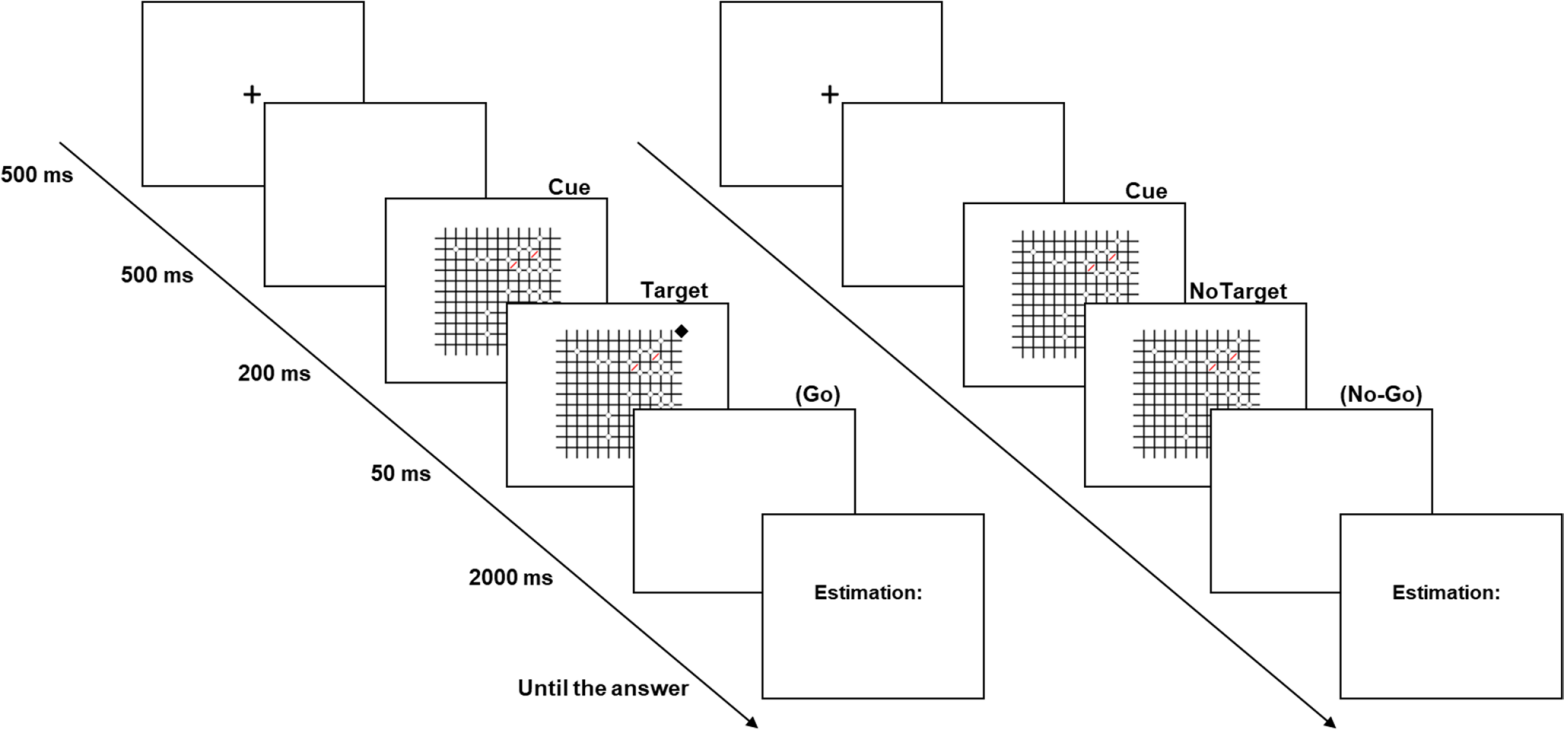
The combined cue-detection & estimation task. Participants performed a dual task: (i) a go/no-go detection task, pressing the space bar if a diamond target appeared in a corner, and (ii) a numerosity estimation task, reporting the number of dots in the array. Each trial began with a fixation cross, followed by the stimulus (250 ms), with the target (when present) displayed for 50 ms. After detection, participants entered their estimate on the keypad

Each trial began with a fixation cross (500 ms), followed by a blank screen (500 ms). The stimulus array was then presented for 250 ms. At 200 ms after onset, a diamond target could appear for 50 ms in one of the four corners of the stimulus. When the target appeared, participants had a fixed response window of 2 s to press the space bar with their left hand. This time window was identical for go and no-go trials, regardless of whether a response was made. Immediately after the detection phase, a visual cue (“Estimation:”) prompted participants to enter their estimate of the numerosity on the numerical keypad using their right hand. After each estimation, participants pressed the space bar to proceed to the next trial (Fig. 2).

The experimental phase consisted of 256 trials, with a self-paced break after half of the trials. The entire session lasted approximately 35 min.

## Results

### Estimation task

#### Go vs. No-Go trials overall analysis

**Fig. 3:**
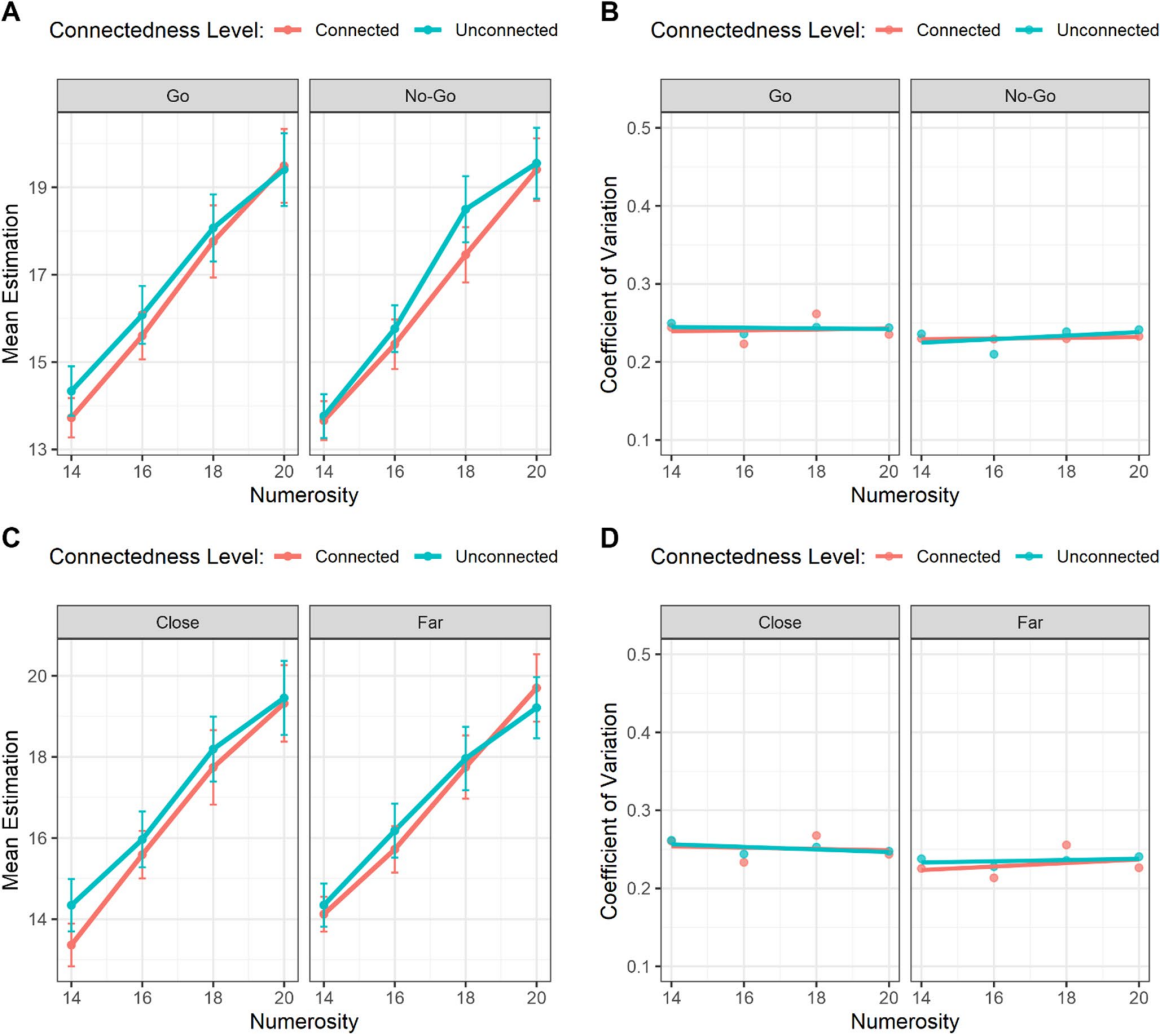
**(A)** Mean estimation as a function of the target Numerosity, the Connectedness Level and the Trial type. **(B)** Mean coefficient of variation as a function of the target Numerosity, the Connectedness Level and the Trial type. **(C)** Mean estimation as a function of the target Numerosity, the Connectedness Level and the Target Position. **(D)** Mean coefficient of variation as a function of the target Numerosity, the Connectedness Level and the Target Position. Bars represent ± 1 standard error of the mean (SEM)

Data analysis was performed with R/R-Studio (2018, v. 3.6.2; http://www.rstudio.com/) software. Individual subjective estimations were examined for the presence of possible aberrant responses (e.g., errors of typing, unrealistic estimations, etc.). To exclude extreme values, we applied an outlier removal procedure based on Weber’s law. For each trial, an expected variability was computed as a function of numerosity (σ = *w* × *Numerosity*), where *w* represents the Weber fraction. Upper and lower cutoffs were then defined as the target *Numerosity* ± k × σ, with k = 2.5 (number of standard deviations) and *w =* 0.18 (average Weber fraction calculated by Anobile et al., 2014) determining the tolerance range. Input values falling outside these bounds were discarded, thus ensuring that only responses consistent with the expected scalar variability were retained for further analyses. A repeated-measures ANOVA with Trial type (Go/No-Go), Connectedness (connected/unconnected), and Numerosity (14, 16, 18, 20) as within-subject factors revealed a robust main effect of Numerosity, *F*(3, 54) = 131.41, *p* <.001, η²ₚ = 0.88. Mauchly’s test indicated a violation of sphericity for this factor, therefore Greenhouse–Geisser correction was applied (ε = 0.50), which confirmed the significance of the effect, *p* <.001. Crucially, there was also a significant main effect of Connectedness, *F*(1, 18) = 12.25, *p* =.003, η²ₚ = 0.41, with connected arrays being systematically underestimated compared to unconnected ones. The main effect of Trial type was not significant, *F*(1, 18) = 1.16, *p* =.296, η²ₚ = 0.06. None of the two- or three-way interactions reached significance (all *ps* > 0.10), including Trial type × Numerosity (*F*(3, 54) = 1.42, *p* =.247, η²ₚ = 0.07), Connectedness × Numerosity (*F*(3, 54) = 1.87, *p* =.146, η²ₚ = 0.09), and the three-way interaction (*F*(3, 54) = 2.18, *p* =.100, η²ₚ = 0.11, Fig. 3A). Thus, the connectedness effect emerged consistently across numerosities and independently of the go/no-go detection task.

To investigate *scalar variability*, a typical behavioral signature of numerosity estimation, a multiple linear regression was conducted to examine the effects of Numerosity, Connectedness, and Trial Type, as well as their interactions, on the Coefficient of Variation (CoV), used as an estimate of the Weber fraction (e.g., Halberda & Odic, 2014). Following Halberda and Odic (2014), the CoV was computed as the ratio between the standard deviation (SD) and the relative mean estimation for each numerosity condition. A slope not significantly different from zero for the predictors would indicate a stable CoV, in line with the Weber’s Law.

The overall model was not significant, *F*(7, 8) = 0.52, *p* =.80, and explained a limited proportion of variance in the outcome (*R*² = 0.31, adj. *R*² = −0.29). Examination of individual predictors revealed that none of the main effects or interactions reached statistical significance. Specifically, Numerosity (β = 0.00059, *p* =.85), Connectedness (β = 0.0197, *p* =.79), and Trial Type (β = −0.0084, *p* =.91) did not significantly predict the dependent measure. Similarly, all two-way and the three-way interactions were non-significant (all *p* >.60, Fig. 3B). These results indicate that, within the tested conditions, none of the factors or their interactions had a detectable effect on the CoV.

#### Go trials only analysis

We further examined subjective estimations for Go trials only, in function of the target distance. Then, we ran a repeated-measures ANOVA (2 × 2 × 4) with Target Position (far/close), Connectedness (connected/unconnected) and Numerosity (14, 16, 18, 20) as within-subject factors and the subjective mean estimation as dependent variable. The results revealed a robust main effect of Numerosity, *F*(3, 54) = 104.34, ε = 0.596, *p* <.001, η²ₚ = 0.85. A significant main effect of Connectedness was also observed, *F*(1, 18) = 4.63, *p* =.045, η²ₚ = 0.21, suggesting that connected arrays were systematically underestimated compared to unconnected ones. In contrast, the main effect of Target Position was not significant, *F*(1, 18) = 0.86, *p* =.367, η²ₚ = 0.05. None of the interaction effects reached significance (all *p*s > 0.20, Fig. 3C).

A multiple linear regression was conducted to examine the effects of Numerosity, Connectedness, and Target Position, as well as their interactions, on the CoV. The overall model was not significant, *F*(7, 8) = 1.04, *p* =.475, indicating that the predictors explained little variance in CoV (*R*² = 0.48, adj. *R*² = 0.02). None of the predictors or interactions reached significance (all *p*s > 0.38, Fig. 3D). In sum, these results replicate the results of the overall analysis.

### Detection task

To further examine potential differences in covert attentional demands between connected and unconnected arrays, we conducted a 2 × 2 × 4 repeated-measures ANOVA on RTs for correct Go-Trial responses (errors discarded: 2.17%), with Target Position (far/close), Connectedness (connected/unconnected), and Numerosity (14, 16, 18, 20) as within-subject factors.

**Fig. 4:**
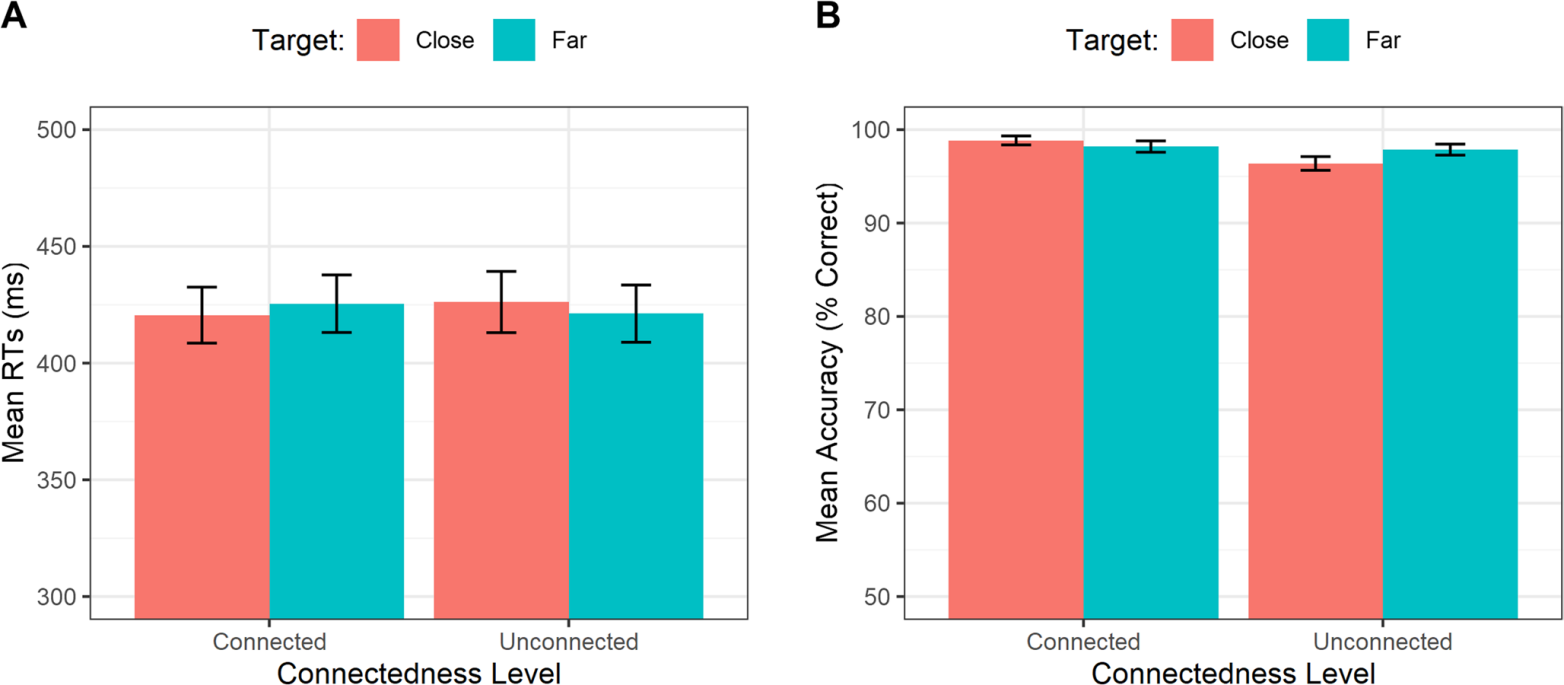
**(A)** Mean RTs as a function of the Connectedness Level and the Target Position. **(B)** Mean Accuracy as a function of the Connectedness Level and the Target Position. Bars represent ± 1 standard error of the mean (SEM)

The ANOVA of mean RTs revealed no significant main effects of Target Position, *F*(1, 18) = 0.00003, *p* =.996, η²ₚ = 0.001, Connectedness, *F*(1, 18) = 0.010, *p* =.920, η²ₚ= 0.001, or Numerosity, *F*(3, 54) = 0.456, *p* =.714, η²ₚ = 0.025. None of the interactions reached significance: Target Position × Connectedness, *F*(1, 18) = 0.787, *p* =.387, η²ₚ = 0.042; Target Position × Numerosity, *F*(3, 54) = 1.324, *p* =.276, η²ₚ = 0.069; Connectedness × Numerosity, *F*(3, 54) = 2.727, *p* =.053, η²ₚ = 0.132; Target Position × Connectedness × Numerosity, *F*(3, 54) = 1.118, *p* =.350, η²ₚ = 0.058, Fig. 4A.

A further ANOVA with the same within factors was ran for the mean Accuracy in the Go-Trials only. The ANOVA of mean Accuracy revealed no significant main effects of Target Position, *F*(1, 18) = 0.411, *p* =.530, η²ₚ = 0.022, Connectedness, *F*(1, 18) = 3.119, *p* =.094, η²ₚ = 0.148, or Numerosity, *F*(3, 54) = 0.587, *p* =.626, η²ₚ = 0.032. No significant interactions emerged: Target Position × Connectedness, *F*(1, 18) = 3.195, *p* =.091, η²ₚ = 0.151; Target Position × Numerosity, *F*(3, 54) = 0.394, *p* =.758, η²ₚ = 0.021; Connectedness × Numerosity, *F*(3, 54) = 1.466, *p* =.234, η²ₚ = 0.075; Target Position × Connectedness × Numerosity, *F*(3, 54) = 0.072, *p* =.975, η²ₚ = 0.004, Fig. 4B.

Additional Bayesian analyses were conducted to quantify the evidence in favor of the null hypotheses. In sum, we found a genuine lack of effects. These secondary analyses are reported in the Supplementary Materials.

## Discussion

The present study examined whether the well-documented underestimation of numerosity in displays containing connected items (or the *connectedness illusion*) is attributable to covert attentional biases rather than to perceptual grouping mechanisms. By integrating a detection task modeled after Posner’s (1980) spatial cueing paradigm with a standard numerosity estimation task, we tested the hypothesis that connected objects might preferentially capture attention, thereby altering performance independently of perceptual grouping.

The results were straightforward: in the estimation task, participants consistently underestimated the numerosity of connected arrays, replicating the classic connectedness effect (e.g., Adriano et al., 2021, 2022; Anobile et al., 2017; Fornaciai & Park, 2018; Franconeri et al., 2009; He et al., 2009, 2015; Pomè et al., 2022). Furthermore, in line with the psychophysical models of numerosity representation (e.g., Whalen et al., 1999), mean estimates and response variability linearly increased with the target numerosity (e.g., scalar variability), resulting in a constant Coefficient of Variation across the numerosity range, trial type and connectedness conditions, as predicted by the Weber’s Law.

Crucially, in the detection task, the performance was unaffected by object connectedness or spatial congruency. This pattern of findings strongly suggests that the connectedness effect is not an attentional artifact. If attentional capture had played a role, targets appearing in the corner close to connected-object cues (or red lines) should have been detected more efficiently than those appearing farther away. That is, detection performance should have suffered in the presence of targets appearing far from connected items due to the automatic redeployment of attentional resources (attentional capture) triggered by multi-part objects. However, neither pattern was observed. Instead, the connectedness underestimation found supports a growing body of evidence indicating that the Approximate Number System (ANS) is shaped by perceptual segmentation mechanisms (e.g., Adriano & Vande Velde, 2025a; Franconeri et al., 2009; He et al., 2009, 2015), which define the units over which numerosity is computed.

One might hypothesize that the absence of cueing effects in the detection task is surprising. Conversely, the absence of cueing effects in the detection task is theoretically meaningful. If connected objects automatically captured covert attention, congruent targets (near the connected lines) should have shown faster reaction times than incongruent targets, yet neither a main effect of congruency nor an interaction with connectedness was observed. Importantly, the detection task preceded the numerosity judgment, ruling out interference from the estimation task. However, it should be considered that our goal was to determine whether connectedness elicits attentional capture within the context of a common numerical task, rather than to directly replicate the standard Posner cueing paradigm. In any case, by using salient red lines and a 50% cue validity, as in standard exogenous cueing paradigms (e.g., Klein, 2009), we provided favorable conditions for triggering attentional capture. The failure of the connected “dumbbell” objects to act as exogenous cues in our task therefore supports our hypothesis that connectedness-induced numerosity underestimation is not caused by spatially biased covert attention.

Another interesting finding of the current study is the absence of a significant difference in numerosity estimation between Go and No-Go trials, and within Go trials, between close and far target conditions. This result aligns with previous evidence suggesting that the Approximate Number System (ANS) operates independently of attentional engagement, particularly when estimating larger quantities. Such findings reinforce the notion that the ANS can support rapid, non-symbolic numerical judgments even under conditions of reduced attentional load (Burr et al., 2010, 2011; Piazza et al., 2011). Indeed, Burr et al. (2010) using a dual task paradigm manipulating attentional load, demonstrated that subitizing—the precise apprehension of small numerosities (e.g., less than 4 items)—breaks down under high attentional load, whereas estimation of larger numerosities remains unaffected. This dissociation indicates that subitizing depends on focused attention, while estimation relies on more automatic or pre-attentive processes.

Furthermore, Pomè et al. (2021) showed that the numerosity underestimation induced by perceptual grouping—specifically, by connecting dot-pairs—is significantly reduced under divided attention, suggesting that grouping mechanisms are attention-dependent. Conversely, the fact that the dual task diminishes the illusion is incompatible with an automatic attentional-capture account, which should be robust to attentional load. Our results further reinforce this interpretation by demonstrating that the observed modulation does not arise from covert attentional capture, but instead reflects a genuine disruption of the object-based segmentation process itself.

Although the present study employed a Posner Go/No-Go paradigm, rather than an explicit dual-task manipulation, both approaches involve variations in attentional engagement. Indeed, in the Posner task, Go trials may impose higher attentional demands than No-Go trials, as participants must detect a cue and respond rapidly pressing a key with the left hand, whereas No-Go trials require only response inhibition since no target is presented. Notably, we also found that within Go trials, numerosity estimation did not differ whether the target appeared close or far from the quadrant containing the connecting lines. Overall, the absence of differences between Go and No-Go (and between close and far trials) in the present study indicates that the Approximate Number System operates robustly even under reduced attentional resources (Burr et al., 2010).

An alternative behavioral pattern, not considered in our study, would have been a main effect of cue location or an interaction between connectedness and cue location on detection-task reaction times without any main effect of cue location or interaction between cue location and connectedness on numerosity estimates. Such a pattern would have indicated that attentional orienting and numerosity underestimation are functionally independent, yet it could also have suggested that spatial attention partially drives numerosity underestimation rather than perceptual grouping. Importantly, our predictions regarding attentional capture were specific to the detection task, and our analysis of cue location in the estimation task serves as an additional control. The actual results provide an even *stronger* dissociation: cue location did not interact with connectedness in the estimation task, and there was no evidence of covert attentional capture in the detection task. These findings therefore rule out the hypothesis that connectedness-induced underestimation is mediated by spatially biased covert attention, while remaining compatible with other roles of attention in numerosity processing (e.g., Pomè et al., 2021). Thus, the target’s spatial position had no effect on performance in either the estimation or detection task, regardless of cue location. Rather, connectedness seems to work as expected, reducing numerosity perception in line with Gestalt laws (Palmer & Rock, 1994), and cannot be explained by covert attentional biases.

According to this view, connected elements are not encoded as separate items but as single grouped objects. This reduces the number of individuated entities available to numerical estimation, leading to systematic underestimation. Such an explanation is consistent with Gestalt principles of perceptual organization (Wagemans et., 2012; Wertheimer, 1923/1950) and recent demonstrations that several grouping cues such as proximity, symmetry, or color similarity can modulate approximate perceived numerosity (e.g., Adriano & Ciccione, 2024; Chakravarthi et al., 2023; He et al., 2009; Franconeri et al., 2009; Maldonado Moscoso et al., 2022).

Our findings extend this literature by showing that the connectedness effect persists even when attentional capture is explicitly measured and ruled out as a potential confound. Hence, beyond ruling out attentional capture as an explanation, our findings strongly contribute to the broader debate about the nature of numerical perception. A central controversy concerns whether the Approximate Number System (ANS) reflects a dedicated perceptual mechanism for extracting numerosity (Burr & Ross, 2008), or whether apparent number sensitivity is instead a by-product of sensitivity to continuous visual dimensions such as density, total area, or spatial frequency content (Gebuis & Reynvoet, 2012a; Leibovich et al., 2017). The persistence of the connectedness effect in our data suggests that underestimation is not reducible to attentional biases but rather arises from perceptual segmentation processes that alter the definition of discrete units over which either numerosity is computed. Crucially, this interpretation is also strongly suggested by the fact that manipulating connectedness did not vary the continuous features in the arrays. More broadly, these results reinforce the notion that visual number perception is therefore a direct read-out of discrete items in a scene, even though the discrete number of objects could be an emergent property of perceptual organization (Anobile et al., 2014; Burr & Ross, 2008; Franconeri et al., 2009). Segmentation and grouping principles appear to act at early perceptual stages, perhaps before or in parallel with attentional mechanisms. Moreover, it remains an open question whether *concurrent* grouping cues in the visual scene (e.g., symmetry, color similarity, connectedness, etc.), as may happen in natural vision, can exert additive effects on numerosity perception (Adriano & Ciccione, 2024; Chakravarthi et al., 2023), and whether such effects are equally resistant to attentional modulation and attentional capture manipulations in Posner-like tasks, as tested in the present study for connectedness only. Neuroimaging and electrophysiological approaches may further clarify whether grouping-induced numerosity underestimation originates from modulations in early visual areas (V1–V4) or from higher-level parietal regions traditionally implicated in numerosity processing (e.g., Harvey et al., 2013). In particular, combining numerosity tasks with population receptive field mapping could help disentangle whether grouping effects arise primarily within early visual cortex or instead reflect computations implemented in parietal areas associated with numerical magnitude representations (e.g., Paul et al., 2022). Finally, the current results open avenues for clinical research. Individuals with dyscalculia may exhibit altered sensitivity to global structure organization, potentially shedding light on the role of perceptual organization in the development and maintenance of numerical cognition (e.g., Castaldi et al., 2022).

In conclusion, the present findings provide strong evidence that the connectedness effect in numerosity perception arises from perceptual grouping rather than covert attentional biases. This supports the broader theoretical claim that object segmentation is a foundational constraint on the ANS and highlights the primacy of perceptual organization in shaping our experience of numerical quantity. Taken together, these findings highlight a crucial boundary condition for theories of numerical cognition: perceptual organization provides the input over which the ANS operates, and this segmentation step is fundamental to how humans perceive and evaluate numerical quantities in their environment.

## Conclusions

The present study demonstrates that the well-known underestimation of numerosity in connected displays cannot be attributed to covert attentional biases. By integrating a spatial cueing paradigm with a numerosity estimation task, we showed that connectedness does not modulate detection performance, thereby ruling out attentional capture as a confounding factor. Instead, the persistence of the connectedness effect strongly supports the view that numerosity perception is constrained by perceptual grouping mechanisms.

These findings reinforce the idea that the Approximate Number System (ANS) operates on the outputs of early segmentation processes, whereby connected elements are encoded as single units rather than distinct items. This highlights perceptual organization as a fundamental step in numerical cognition, shaping the very input over which the ANS computes. More broadly, our results point to object segmentation as a core constraint on number perception, with implications for theories of numerical cognition as well as for future developmental, clinical, and neuroimaging research.

## Data Availability

The datasets generated during the current study are available from the authors upon request.

## Declarations

### Open practice statement

The data for all experiments are available from the authors upon request.

## References

[CR1] Aagten-Murphy, D., & Burr, D. (2016). Adaptation to numerosity requires only brief exposures, and is determined by number of events, not exposure duration. *Journal of Vision*, *16*(10), 1–14. 10.1167/16.10.22 PMC5053365 27580042

[CR2] Adriano, A., & Ciccione, L. (2024). The interplay between spatial and non-spatial grouping cues over approximate number perception. *Attention, Perception, & Psychophysics*. 10.3758/s13414-024-02908-4 10.3758/s13414-024-02908-4 38858304

[CR3] Adriano, A., & Vande Velde, M. (2025a). Number is more than meets the eye: Unveiling segmentation mechanisms in numerosity perception with visual illusions. *Vision Research,**228*, 108547. 39879872 10.1016/j.visres.2025.108547

[CR4] Adriano, A., & Vande Velde, M. (2025b). Numerosity adaptation resists filtering: Insights from an illusory contour paradigm. *Cognitive Psychology*, *160*, 101757. 40829196 10.1016/j.cogpsych.2025.101757

[CR5] Adriano, A., Rinaldi, L., & Girelli, L. (2021). Visual illusions as a tool to hijack numerical perception: Disentangling nonsymbolic number from its continuous visual properties. *Journal of Experimental Psychology: Human Perception and Performance*, *47*(3), 423–441. 33492161 10.1037/xhp0000844

[CR6] Adriano, A., Rinaldi, L., & Girelli, L. (2022). Nonsymbolic numerosity in sets with illusory-contours exploits a context-sensitive, but contrast-insensitive, visual boundary formation process. *Attention, Perception, & Psychophysics,* (1), 16. 10.3758/s13414-021-02378-y 10.3758/s13414-021-02378-y PMC8520761 34658000

[CR7] Agrillo, C., Dadda, M., Serena, G., & Bisazza, A. (2009). Use of number by fish. *PLoS One,**4*(3), Article e4786. 19274079 10.1371/journal.pone.0004786 PMC2650784

[CR8] Agrillo, C., Piffer, L., Bisazza, A., & Butterworth, B. (2012). Evidence for two numerical systems that are similar in humans and guppies. *PLoS One,**7*(2), Article e31923. 22355405 10.1371/journal.pone.0031923 PMC3280231

[CR9] Allik, J., & Tuulmets, T. (1991). Occupancy model of perceived numerosity. *Perception & Psychophysics*, *49*(4), 303–314. 2030927 10.3758/bf03205986

[CR10] Anobile, G., Cicchini, G. M., & Burr, D. C. (2014). Separate mechanisms for perception of numerosity and density. *Psychological Science,**25*(1), 265–270. 24270462 10.1177/0956797613501520

[CR11] Anobile, G., Turi, M., Cicchini, G. M., & Burr, D. C. (2015). Mechanisms for perception of numerosity or texture-density are governed by crowding-like effects. *Journal of Vision,**15*(5), 1–12. 10.1167/15.5.4 PMC4909146 26067522

[CR12] Anobile, G., Cicchini, G. M., & Burr, D. C. (2016). Number as a primary perceptual attribute: A review. *Perception,**45*(1–2), 5–31. 26562858 10.1177/0301006615602599 PMC5040510

[CR13] Anobile, G., Cicchini, G. M., Pomè, A., & Burr, D. C. (2017). Connecting visual objects reduces perceived numerosity and density for sparse but not dense patterns. *Journal of Numerical Cognition*, *3*(2), 133–146.

[CR14] Arrighi, R., Togoli, I., & Burr, D. C. (2014). A generalized sense of number. *Proceedings of the Royal Society B: Biological Sciences,**281*(1797), Article 20141791. 10.1098/rspb.2014.1791 PMC4240988 25377454

[CR15] Aulet, L. S., & Lourenco, S. F. (2023). Visual adaptation reveals multichannel coding for numerosity. *Frontiers in Psychology,**14*, Article 1125925. 37168429 10.3389/fpsyg.2023.1125925 PMC10164939

[CR16] Benson-Amram, S., Heinen, V. K., Dryer, S. L., & Holekamp, K. E. (2011). Numerical assessment and individual call discrimination by wild spotted hyaenas, *Crocuta crocuta*. *Animal Behaviour,**82*(4), 743–752.

[CR17] Brannon, E. M., & Terrace, H. S. (1998). Ordering of the numerosities 1 to 9 by monkeys. *Science,**282*(5389), 746–749. 9784133 10.1126/science.282.5389.746

[CR18] Brannon, E. M., Abbott, S., & Lutz, D. J. (2004). Number bias for the discrimination of large visual sets in infancy. *Cognition,**93*(2), B59–B68. 15147939 10.1016/j.cognition.2004.01.004

[CR19] Brus, J., Heng, J. A., & Polanía, R. (2019). Weber’s law: A mechanistic foundation after two centuries. *Trends in Cognitive Sciences*, *23*(11), 906–908. 31629634 10.1016/j.tics.2019.09.001

[CR20] Burr, D., & Ross, J. (2008). A visual sense of number. *Current Biology*, *18*(6), 425–428. 18342507 10.1016/j.cub.2008.02.052

[CR21] Burr, D. C., Turi, M., & Anobile, G. (2010). Subitizing but not estimation of numerosity requires attentional resources. *Journal of Vision,**10*(6), 1–10. 10.1167/10.6.20 20884569

[CR22] Burr, D. C., Anobile, G., & Turi, M. (2011). Adaptation affects both high and low (subitized) numbers under conditions of high attentional load. *Seeing and Perceiving,**24*(2), 141–150. 21864455 10.1163/187847511X570097

[CR23] Burr, D., Anobile, G., & Arrighi, R. (2025). Number adaptation: Reply. *Cognition,**254*, Article 105870. 39617517 10.1016/j.cognition.2024.105870

[CR24] Castaldi, E., Turi, M., Cicchini, G. M., Gassama, S., & Eger, E. (2022). Reduced 2D form coherence and 3D structure from motion sensitivity in developmental dyscalculia. *Neuropsychologia,**166*, Article 108140. 34990696 10.1016/j.neuropsychologia.2021.108140

[CR25] Castelli, F., Glaser, D. E., & Butterworth, B. (2006). Discrete and analogue quantity processing in the parietal lobe: A functional MRI study. *Proceedings of the National Academy of Sciences*, *103*(12), 4693–4698. 10.1073/pnas.0600444103 PMC1450233 16537401

[CR26] Chakravarthi, R., Nordqvist, A., Poncet, M., & Adamian, N. (2023). Fundamental units of numerosity estimation. *Cognition,**239*, Article 105565. 37487302 10.1016/j.cognition.2023.105565

[CR27] Dakin, S. C., Tibber, M. S., Greenwood, J. A., & Morgan, M. J. (2011). A common visual metric for approximate number and density. *Proceedings of the National Academy of Sciences,**108*(49), 19552–19557. 10.1073/pnas.1113195108 PMC3241748 22106276

[CR28] Dehaene, S. (2003). The neural basis of the Weber–Fechner law: A logarithmic mental number line. *Trends in Cognitive Sciences*, *7*(4), 145–147. 12691758 10.1016/s1364-6613(03)00055-x

[CR29] Dehaene, S., & Changeux, J. P. (1993). Development of elementary numerical abilities: A neuronal model. *Journal of Cognitive Neuroscience,**5*(4), 390–407. 23964915 10.1162/jocn.1993.5.4.390

[CR30] Dehaene, S., Dehaene-Lambertz, G., & Cohen, L. (1998). Abstract representations of numbers in the animal and human brain. *Trends in Neurosciences*, *21*(8), 355–361. 9720604 10.1016/s0166-2236(98)01263-6

[CR31] DeWind, N. K., Park, J., Woldorff, M. G., & Brannon, E. M. (2019). Numerical encoding in early visual cortex. *Cortex,**114*, 76–89. 29983159 10.1016/j.cortex.2018.03.027 PMC6170729

[CR32] Ditz, H. M., & Nieder, A. (2015). Neurons selective to the number of visual items in the corvid songbird endbrain. *Proceedings of the National Academy of Sciences,**112*(25), 7827–7832. 10.1073/pnas.1504245112 PMC4485087 26056278

[CR33] Durgin, F. H. (2008). Texture density adaptation and visual number revisited. *Current Biology,**18*(18), R855–R856. 18812077 10.1016/j.cub.2008.07.053

[CR34] Faul, F., Erdfelder, E., Buchner, A., & Lang, A. G. (2009). Statistical power analyses using G* power 3.1: Tests for correlation and regression analyses. *Behavior Research Methods*, *41*(4), 1149–1160. 19897823 10.3758/BRM.41.4.1149

[CR35] Fornaciai, M., & Park, J. (2018). Early numerosity encoding in visual cortex is not sufficient for the representation of numerical magnitude. *Journal of Cognitive Neuroscience*, *30*(12), 1788–1802. 30063175 10.1162/jocn_a_01320

[CR36] Fornaciai, M., Brannon, E. M., Woldorff, M. G., & Park, J. (2017). Numerosity processing in early visual cortex. *NeuroImage,**157*, 429–438. 28583882 10.1016/j.neuroimage.2017.05.069 PMC6697050

[CR37] Franconeri, S. L., Bemis, D. K., & Alvarez, G. A. (2009). Number estimation relies on a set of segmented objects. *Cognition,**113*(1), 1–13. 19647817 10.1016/j.cognition.2009.07.002

[CR38] Gebuis, T., & Reynvoet, B. (2012a). The interplay between nonsymbolic number and its continuous visual properties. *Journal of Experimental Psychology: General*, *141*(4), 642–648. 22082115 10.1037/a0026218

[CR39] Gebuis, T., & Reynvoet, B. (2012b). The role of visual information in numerosity estimation. *PLoS One,**7*(5), e37426. 22616007 10.1371/journal.pone.0037426 PMC3355123

[CR40] Gebuis, T., & Reynvoet, B. (2012c). Continuous visual properties explain neural responses to nonsymbolic number. *Psychophysiology*, *49*(11), 1649–1659. 10.1111/j.1469-8986.2012.01461.x 23046492

[CR41] Gebuis, T., Kadosh, R. C., & Gevers, W. (2016). Sensory-integration system rather than approximate number system underlies numerosity processing: A critical review. *Acta Psychologica,**171*, 17–35. 27640140 10.1016/j.actpsy.2016.09.003

[CR42] Halberda, J., & Odic, D. (2014). The precision and internal confidence of our approximate number thoughts. In D. C. Geary, D. Berch, & K. Koepke (Eds.), *Evolutionary origins and early development of number processing* (pp. 305–333). Academic Press.

[CR43] Harvey, B. M., Klein, B. P., Petridou, N., & Dumoulin, S. O. (2013). Topographic representation of numerosity in the human parietal cortex. *Science,**341*(6150), 1123–1126. 24009396 10.1126/science.1239052

[CR44] He, L., Zhang, J., Zhou, T., & Chen, L. (2009). Connectedness affects dot numerosity judgment: Implications for configural processing. *Psychonomic Bulletin & Review,**16*(3), 509–517. 19451377 10.3758/PBR.16.3.509

[CR45] He, L., Zhou, K., Zhou, T., He, S., & Chen, L. (2015). Topology-defined units in numerosity perception. *Proceedings of the National Academy of Sciences*, *112*(41), E5647–E5655. 10.1073/pnas.1512408112 PMC4611617 26417075

[CR46] Hurewitz, F., Gelman, R., & Schnitzer, B. (2006). Sometimes area counts more than number. *Proceedings of the National Academy of Sciences*, *103*(51), 19599–19604. 10.1073/pnas.0609485103 PMC1748271 17159143

[CR47] Katzin, N., Katzin, D., Rosén, A., Henik, A., & Salti, M. (2020). Putting the world in mind: The case of mental representation of quantity. *Cognition,**195*, Article 104088. 31734547 10.1016/j.cognition.2019.104088

[CR48] Kirjakovski, A., & Matsumoto, E. (2016). Numerosity underestimation in sets with illusory contours. *Vision Research*, *122*, 34–42. 27038561 10.1016/j.visres.2016.03.005

[CR49] Klein, R. (2009). On the control of attention. *Canadian Journal of Experimental Psychology = Revue Canadienne De Psychologie Experimentale,**63*(3), 240–252. 19739907 10.1037/a0015807

[CR50] Leibovich, T., Katzin, N., Harel, M., & Henik, A. (2017). From “sense of number” to “sense of magnitude”: The role of continuous magnitudes in numerical cognition. *Behavioral and Brain Sciences,**40*, Article e164. 27530053 10.1017/S0140525X16000960

[CR51] Maldonado Moscoso, P. A., Anobile, G., Burr, D. C., Arrighi, R., & Castaldi, E. (2022). Symmetry as a grouping cue for numerosity perception. *Scientific Reports,**12*(1), Article 14418. 36002617 10.1038/s41598-022-18386-3 PMC9402546

[CR52] Morgan, M. J., Raphael, S., Tibber, M. S., & Dakin, S. C. (2014). A texture-processing model of the ‘visual sense of number.’ *Proceedings of the Royal Society B: Biological Sciences,**281*(1790), Article 20141137. 10.1098/rspb.2014.1137 PMC4123707 25030988

[CR53] Nieder, A. (2016). The neuronal code for number. *Nature Reviews Neuroscience*, *17*(6), 366–382. 27150407 10.1038/nrn.2016.40

[CR54] Nieder, A., & Miller, E. K. (2004). A parieto-frontal network for visual numerical information in the monkey. *Proceedings of the National Academy of Sciences,**101*(19), 7457–7462. 10.1073/pnas.0402239101 PMC409940 15123797

[CR55] Palmer, S., & Rock, I. (1994). Rethinking perceptual organization: The role of uniform connectedness. *Psychonomic Bulletin & Review*, *1*(1), 29–55. 24203413 10.3758/BF03200760

[CR56] Park, J., DeWind, N. K., Woldorff, M. G., & Brannon, E. M. (2015). Rapid and direct encoding of numerosity in the visual stream. *Cerebral Cortex,**26*(2), 748–763. 25715283 10.1093/cercor/bhv017 PMC4712802

[CR57] Paul, J. M., van Ackooij, M., Ten Cate, T. C., & Harvey, B. M. (2022). Numerosity tuning in human association cortices and local image contrast representations in early visual cortex. *Nature Communications,**13*(1), Article 1340. 35292648 10.1038/s41467-022-29030-z PMC8924234

[CR58] Peirce, J. W. (2007). PsychoPy-psychophysics software in Python. *Journal of Neuroscience Methods,**162*(1–2), 8–13. 17254636 10.1016/j.jneumeth.2006.11.017 PMC2018741

[CR59] Perdue, B. M., Talbot, C. F., Stone, A. M., & Beran, M. J. (2012). Putting the elephant back in the herd: Elephant relative quantity judgments match those of other species. *Animal Cognition,**15*(5), 955–961. 22692435 10.1007/s10071-012-0521-y

[CR60] Piantadosi, S. T., & Cantlon, J. F. (2017). True numerical cognition in the wild. *Psychological Science*, *28*(4), 462–469. 28406373 10.1177/0956797616686862 PMC5407312

[CR61] Piazza, M., Izard, V., Pinel, P., Le Bihan, D., & Dehaene, S. (2004). Tuning curves for approximate numerosity in the human intraparietal sulcus. *Neuron,**44*(3), 547–555. 15504333 10.1016/j.neuron.2004.10.014

[CR62] Piazza, M., Fumarola, A., Chinello, A., & Melcher, D. (2011). Subitizing reflects visuo-spatial object individuation capacity. *Cognition*, *121*(1), 147–153. 21679934 10.1016/j.cognition.2011.05.007

[CR63] Picon, E., Dramkin, D., & Odic, D. (2019). Visual illusions help reveal the primitives of number perception. *Journal of Experimental Psychology: General*, *148*(10), 1675–1687. 30730194 10.1037/xge0000553

[CR64] Pomè, A., Anobile, G., Cicchini, G. M., Scabia, A., & Burr, D. C. (2019). Higher attentional costs for numerosity estimation at high densities. *Attention, Perception & Psychophysics,**81*(8), 2604–2611. 10.3758/s13414-019-01831-3 PMC6856040 31407272

[CR65] Pomè, A., Caponi, C., & Burr, D. C. (2021). The grouping-induced numerosity illusion is attention-dependent. *Frontiers in Human Neuroscience,**15*, Article 745188. 34690725 10.3389/fnhum.2021.745188 PMC8528175

[CR66] Pomè, A., Caponi, C., & Burr, D. C. (2022). Grouping-induced numerosity biases vary with autistic-like personality traits. *Journal of Autism and Developmental Disorders*, *52*(3), 1326–1333. 33909210 10.1007/s10803-021-05029-1 PMC8854316

[CR67] Portley, M., & Durgin, F. H. (2019). The second number-estimation elbow: Are visual numbers greater than 20 evaluated differently? *Attention Perception & Psychophysics*, *81*(5), 1512–1521. 10.3758/s13414-019-01804-6 31267478

[CR68] Posner, M. I. (1980). Orienting of attention. *Quarterly Journal of Experimental Psychology,**32*(1), 3–25. 7367577 10.1080/00335558008248231

[CR69] Revkin, S. K., Piazza, M., Izard, V., Cohen, L., & Dehaene, S. (2008). Does subitizing reflect numerical estimation? *Psychological Science*, *19*(6), 607–614. 18578852 10.1111/j.1467-9280.2008.02130.x

[CR70] RStudio, T., & RStudio (2018). Inc., Boston, MA URL http://www.rstudio.com/.

[CR71] Stoianov, I., & Zorzi, M. (2012). Emergence of a ‘visual number sense’ in hierarchical generative models. *Nature Neuroscience*, *15*(2), 194–196. 22231428 10.1038/nn.2996

[CR72] Thompson, P., & Burr, D. (2009). Visual aftereffects. *Current Biology*, *19*(1), R11–R14. 19138580 10.1016/j.cub.2008.10.014

[CR73] Van Rinsveld, A., Guillaume, M., Kohler, P. J., Schiltz, C., Gevers, W., & Content, A. (2020). The neural signature of numerosity by separating numerical and continuous magnitude extraction in visual cortex with frequency-tagged EEG. *Proceedings of the National Academy of Sciences,**117*(11), 5726–5732. 10.1073/pnas.1917849117 PMC7084102 32123113

[CR74] Verguts, T., & Fias, W. (2004). Representation of number in animals and humans: A neural model. *Journal of Cognitive Neuroscience*, *16*(9), 1493–1504. 15601514 10.1162/0898929042568497

[CR75] Wagemans, J., Elder, J. H., Kubovy, M., Palmer, S. E., Peterson, M. A., Singh, M., & von der Heydt, R. (2012). A century of Gestalt psychology in visual perception: I. Perceptual grouping and figure–ground organization. *Psychological Bulletin,**138*(6), 1172–1217. 22845751 10.1037/a0029333 PMC3482144

[CR76] Wertheimer, M. (1923/1950). Investigations in gestalt theory. In W. D. Ellis (Ed.), *A source book of gestalt psychology* (pp. 71–88). Routledge & Kegan Paul. (Original work published 1923).

[CR77] Whalen, J., Gallistel, C. R., & Gelman, R. (1999). Nonverbal counting in humans: The psychophysics of number representation. *Psychological Science*, *10*(2), 130–137.

[CR78] Xu, F., & Spelke, E. S. (2000). Large number discrimination in 6-month-old infants. *Cognition*, *74*(1), B1–B11. 10594312 10.1016/s0010-0277(99)00066-9

[CR79] Xu, F., Spelke, E. S., & Goddard, S. (2005). Number sense in human infants. *Developmental Science*, *8*(1), 88–101. 15647069 10.1111/j.1467-7687.2005.00395.x

[CR80] Yousif, S. R., Clarke, S., & Brannon, E. M. (2024). Number adaptation: A critical look. *Cognition,**249*, Article 105813. 38820687 10.1016/j.cognition.2024.105813

[CR81] Yousif, S. R., Clarke, S., & Brannon, E. M. (2025). Seven reasons to (still) doubt the existence of number adaptation: A rebuttal to Burr et al. and Durgin. *Cognition,**254*, Article 105939. 39317022 10.1016/j.cognition.2024.105939

